# Detectability Analysis of Road Vehicles in Radarsat-2 Fully Polarimetric SAR Images for Traffic Monitoring

**DOI:** 10.3390/s17020298

**Published:** 2017-02-06

**Authors:** Bo Zhang, Chao Wang, Hong Zhang, Fan Wu, Yi-Xian Tang

**Affiliations:** Key Laboratory of Digital Earth Science, Institute of Remote Sensing and Digital Earth, Chinese Academy of Sciences, Beijing 100094, China; zhangbo@radi.ac.cn (B.Z.); zhanghong@radi.ac.cn (H.Z.); wufan@radi.ac.cn (F.W.); tangyx@radi.ac.cn (Y.-X.T.)

**Keywords:** Synthetic Aperture Radar, vehicle detectability, Radarsat-2 satellite, traffic monitoring

## Abstract

By acquiring information over a wide area regardless of weather conditions and solar illumination, space-borne Synthetic Aperture Radar (SAR) has the potential to be a promising application for traffic monitoring. However, the backscatter character of a vehicle in a SAR image is unstable and varies with image parameters, such as aspect and incidence angle. To investigate vehicle detectability in SAR images for traffic monitoring applications, images of four common types of vehicles in China were acquired using the fully polarimetric (FP) SAR of Radarsat-2 in our experiments. Methods for measuring a vehicle’s aspect angle and backscatter intensity are introduced. The experimental FP SAR images are used to analyze the detectability, which is affected by factors such as vehicle size, vehicle shape, and aspect angle. Moreover, a new metric to improve vehicle detectability in FP SAR images is proposed and compared with the well-known intensity metric. The experimental results show that shape is a crucial factor in affecting the backscatter intensity of vehicles, which also oscillates with varying aspect angle. If the size of a vehicle is smaller than the SAR image resolution, using the intensity metric would result in low detectability. However, it could be improved in an FP SAR image by using the proposed metric. Compared with the intensity metric, the overall detectability is improved from 72% to 90% in our experiments. Therefore, this study indicates that FP SAR images have the ability to detect stationary vehicles on the road and are meaningful for traffic monitoring.

## 1. Introduction

Compared with optic sensors, Synthetic Aperture Radar (SAR) has the advantage of being able to image under all weather conditions and is independent of solar illumination, so it is very attractive for surveillance applications. SAR images have been used extensively for marine applications, such as ship detection and classification [1,2]. Recently, recognition of civilian aircrafts has been demonstrated with SAR images at a 1-meter resolution [3]. Monitoring of road vehicles in wide areas is another potential application of SAR that could be useful for transportation authorities. For example, in bad weather conditions, such as rain, snow, or fog, vehicles move slowly, and traffic becomes congested. When this occurs in a wide area, space-borne SAR may be the best choice for monitoring traffic because it can effectively capture a snapshot of the region at low cost regardless of the weather condition.

Many scholars have carried out meaningful work regarding the application of SAR images for vehicle monitoring. For example, a schedule for an operational satellite-based traffic monitoring system was designed by the German Aerospace Center (DLR) [4]. Beginning with conceptual studies for the TerraSAR-X dual receive antenna beginning in 2003 [5], a series of research results have been published. Their achievements can be mainly classified into four categories: quantitative analysis of the influence of target motion and velocity on displaced and blurred SAR image azimuth direction [6,7,8]; analysis of parking vehicle characteristics based on SAR simulation [8] and real air-borne experimental E-SAR (The Experimental airborne SAR System of DLR) data [9,10,11]; target detector design including a Displaced Phase Centre Antenna detector, an along track interferometry (ATI) constant false alarm rate detector, an frequency modulation (FM) rate variation detector, and the integration of an a-prior knowledge detector [8]; and experimental verification and performance analysis conducted based on air-borne E-SAR data [9,10,11].

Recently, miniSAR images with a very high resolution of 0.1 m × 0.1 m were presented and used to detect cars by adapted constant false rate (CFAR) detectors [12,13]. Another new image mode, named wide-angle SAR, was reported and the results show that the vehicle detection performance is improved with its increased azimuth extent [14]. Compared with the advanced air-borne miniSAR and wide-angle SAR, space-borne SAR in orbit now has limited image resolution and azimuth extent. If this space-borne SAR image is applied to vehicle detection, more issues should be considered, such as how small-sized vehicles can be confidently detected and whether fully polarimetric (FP) images can improve the probability of detection (hereinafter referred to as detectability). To address these problems, images of four common types of vehicles in China were acquired using the fully polarimetric (FP) SAR of Radarsat-2, which were then used to analyze the detectability of vehicles. Compared with the preceding literature [10,11,12,13,14,15], the novelties of this paper lie in the following two aspects. First, more factors that affect the detectability of vehicles in space-borne SAR images are taken into account, for e.g., the vehicle’s size, shape and aspect angles. As a result, the importance of these factors is quantitatively analyzed and compared according to their effect on detectability. Moreover, to demonstrate the potential application of traffic monitoring with fine resolution of FP SAR images, a new metric to improve detectability is proposed. This metric is based on the technique of polarimetric decomposition and its performance is validated by comparison with the traditional intensity metric.

The remainder of this paper is organized as follows: Section 2 introduces experimental Radarsat-2 FP SAR images and features of four types of road vehicles. Section 3 describes the calculation methods for the detectability of road vehicles. Experiments and discussion of the influence on vehicle detectability are presented in Section 4 and Section 5, respectively. Finally, in Section 6, conclusions are drawn regarding the potential application of these results for traffic monitoring.

## 2. SAR Images and Road Vehicles

### 2.1. Radarsat-2 FP SAR Images

The Radarsat-2 satellite can offer commercial products at C-band with single and full polarization. Considering the real-world practicality of their applications, FP products would be a good compromise in resolution, polarization, and cost. FP products contain additional polarimetric information at 10 m resolution and can be purchased at an acceptable price. To date, we have collected four types of single-look FP images in our experiments. The main details of the nominal parameters for each type of SAR image are listed in Table 1.

### 2.2. Road Vehicles

Four types of vehicles, medium bus, medium truck, larger truck, and tank truck, were used in our experiments. These vehicles are the most common means of transport in China. Medium buses are used for inter-city passenger transport. Medium trucks are usually used for short-distance transport of bulk goods, such as vegetables, fruits, and articles for daily use. Larger trucks are mainly used for long-distance transport. Medium and larger trucks have the same canvas cover, which covers the body when they are loaded. From the viewpoint of shape, medium buses, medium trucks, and larger trucks are similar and can be simply represented as cuboids. The last type of vehicle is the larger tank truck, which is used to carry liquid loads, and its shape is dominated by its cylindrical tank. The size details of the vehicles are listed in Table 2, and their in-sites photos are given in Section 4.

## 3. Methods

### 3.1. Aspect Angle Measurement

**Figure 1:**
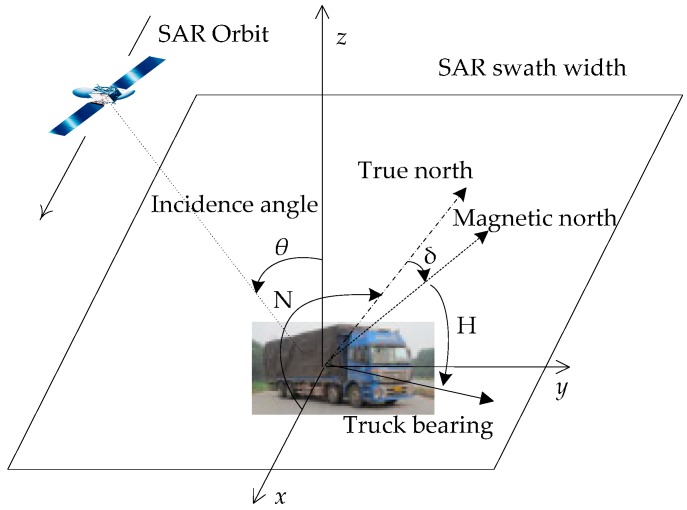
Measurement of aspect angle and local incidence angle at the defined Cartesian coordinates. SAR stands for Synthetic Aperture Radar.

According to the principle of radar backscattering, the backscatter intensity of a vehicle is mainly affected by the geometric relationship between the vehicle and the SAR sensor. Because the roads are usually flat, three axes (yaw, pitch, and roll) are used to describe the vehicle’s attitude, and these can be reduced to a simplified parameter, i.e., aspect angle. Then, the geometric relationship is decomposed into the aspect angle of the vehicle and the incidence angle of the SAR sensor. For a given imaging mode of SAR, the nominal incidence angle is pre-known. However, the aspect angle of the vehicle may vary, so it must be predetermined and measured in each experiment. To define the aspect angle and local incidence angle, Cartesian coordinates are assigned for each vehicle, as shown in Figure 1.

The coordinate origin is set at the center of the vehicle, the *x*-direction in the horizontal plane parallels the SAR orbit direction, the *z*-direction is normal to the horizontal plane, and the *y*-direction constitutes a right-handed coordinate system. By regarding the *x*-direction as the original direction 0° and considering the clockwise direction as positive, the aspect angle is defined as the angles from the SAR orbit direction (*x*-direction) to the vehicle bearing direction. Thus, the range of aspect angle is [0°, 360°). The aspect angle ∠S is measured with a portable compass and calculated using the following formula:

(1) ∠S=[N+(H+δ)]%360

where H is the angle from the magnetic north direction to the vehicle’s bearing; δ represents the local magnetic declination; N is the angle from the *x*-direction to true north; and the symbol % is a remainder operator to maintain the calculated result in the range of [0°, 360°). If the orientation of the aspect angle is not considered, the aspect angle can be simply substituted by the intersection angles; a detailed definition of this is given in Section 5. Considering the propagation of error from the operation of the compass, the accuracy of the calculated aspect angle can be within ±1°.

The local incidence angle is defined as the angle between the line of sight of the SAR and the local normal *z*-direction. It can be calculated using the position vector composed of the SAR satellite and the vehicle. The SAR satellite position can be obtained by interpolating the ephemeris, and the vehicle position can be resolved by its image coordinates. Details of the process for position calculation are included in the Range-Doppler model for SAR image geocoding [16].

### 3.2. Measurement of Radar Cross Section (RCS)

The vehicles’ backscatter intensity is represented by Radar Cross Section (RCS, *σ*) in the SAR images and calculated with the following formula [11]:

(2) $$\sigma =\int_{-\infty }^{+\infty }\int_{-\infty }^{+\infty }\sigma_{0}\left(x,y\right)dxdy\approx \Delta x\cdot \Delta y\cdot \sum_{c,t}\sigma_{0}\left(c,t\right)$$

where Δx⋅Δy represents the ground area of one pixel in the SAR image and $\sum_{c,t}\sigma_{0}\left(c,t\right)$ stands for the area integral within the vehicle contour at the center position of (c,t). For commercial Radarsat-2 images, $\sigma_{0}$ of each pixel can be directly obtained from digital number (DN) values using the radiant correction parameters given in the lutSigma.xml product file.

In practical applications, the performance of an object detector such as the CFAR detector is determined by the signal-to-noise ratio (S/N) [17]. To reduce the influence of speckle noise and enhance the intensity signal of the vehicle, the FP SAR image is usually transformed into a total scattered power (SPAN) image and is then used to evaluate the detectability for vehicles. The SPAN image is combined as follows [18]:

(3) $$\mathrm{SPAN}={\left|s_{hh}\right|}^{2}+{\left|s_{hv}\right|}^{2}+{\left|s_{vh}\right|}^{2}+{\left|s_{vv}\right|}^{2}$$

where $s_{ij}$ denotes each channel of the FP SAR image. It can be seen that four channels are superimposed in this formula, indicating the incoherence accumulation at each pixel value. Therefore, the intrinsic speckle would be deeply suppressed in the resulting of SPAN image. Considering the change of aspect angle, a vehicle in the SAR image may occupy one or more pixels. Therefore, once the CFAR detector is applied to the SPAN image, the pixel holding the maximum value in a vehicle image chip is considered the center of the vehicle target. Therefore, this maximum value represents the vehicle’s RCS and is used as the intensity metric for the backscatter characteristic and detectability analysis in the following discussion.

To obtain the road backscatter intensity, some patches in the road between the trucks should be extracted and used to calculate their statistical parameters, such as the mean and standard deviation.

### 3.3. Pauli Decomposition

Pauli decomposition is a well-known technique used for FP SAR image decomposition, which expresses the scattering matrix [S] of an FP SAR image with the so-called Pauli basis ${\left[S\right]}_{a}$, ${\left[S\right]}_{b}$, and ${\left[S\right]}_{c}$ as follows [18]:

(4) $$\left[s\right]=\left[\begin{array}{cc} s_{hh} & s_{hv}\\ s_{vh} & s_{vv} \end{array}\right]=\alpha {\left[S\right]}_{a}+\beta {\left[S\right]}_{b}+\gamma {\left[S\right]}_{c}$$

(5) $$\begin{array}{c} a=\left(s_{hh}+s_{vv}\right)/\sqrt{2}\\ \beta =\left(s_{hh}-s_{vv}\right)/\sqrt{2}\\ \gamma =\sqrt{2}s_{hv} \end{array}$$

where matrix ${\left[S\right]}_{a}$ represents single or odd-bounce scattering; matrix ${\left[S\right]}_{b}$ indicates double or even-bounce scattering, and matrix ${\left[S\right]}_{c}$ represents volume scattering. The ${\left|\alpha \right|}^{2}$, ${\left|\beta \right|}^{2}$, and ${\left|\gamma \right|}^{2}$ correspond to the scattered power of each scattering mechanism. In detail, the power of ${\left|\alpha \right|}^{2}$ is mainly caused by the vehicles’ components, such as trihedral structures and small planes. A dihedral structure composed of the vehicle side and the ground surface contributes to the power of ${\left|\beta \right|}^{2}$. Meanwhile, the power of ${\left|\gamma \right|}^{2}$ is due to the mechanism of random scattering, which is related to the random surface roughness or other complex structures of the vehicle. As a vehicle turns, its attributes, including local structure and shape, that face the SAR sensor change. Consequently, the backscattering power of each Pauli component varies. This phenomenon is based on the grounds that the vehicle is heterogeneous and causes variable intensity as it turns. In contrast, the road surface is homogeneous, so its backscattering is stable and dominated by diffuse scatterings as a result of Pauli decomposition. When Pauli decomposition is applied to a single-look FP SAR image, filters such as boxcar and Lee are usually used as standard processing to suppress the intrinsic speckles [18].

### 3.4. Detectability Calculation with Different Metrics

The relationship between the probability of detection, i.e., detectability ($P_{d}$) and the false alarm rate $(P_{\mathrm{fa}}$) is related to the factor of S/N [17], which is expressed as

(6) S/N=A+0.12AB+1.7B

where $A=\ln\left(0.62/P_{\mathrm{fa}}\right),$ $B=\ln\left(P_{d}/(1-P_{d})\right)$, and ln(⋅) is the natural logarithm operator. $P_{\mathrm{fa}}$ can be calculated by the formula

(7) $$P_{\mathrm{fa}}=1-\int_{-\infty }^{x_{0}}f\left(x\right)dx$$

where f(x) denotes the probability distribution function of the background clutter in SAR images and $x_{0}$ is the threshold value. If one pixel value is higher than $x_{0}$, it will be extracted from the background as the target.

According to Formula (6), S/N is a crucial factor to achieve a high value of $P_{d}$ because the value $P_{\mathrm{fa}}$ is always prefixed and set as a constant throughout the detection process. In other words, the higher the value of S/N, the higher the value of $P_{d}$. Thus, improving detectability can be achieved by enhancing signal intensity or reducing the power of the background. In the process of calculating the detectability using Formula (6) in SPAN images, signal power represents the vehicle’s RCS and background power corresponds to the mean value of the road’s RCS.

For FP SAR images, a detector can also be applied to any decomposed component or their combinations [19]. The backscatters of the road background are homogeneous and stable, and in contrast, the vehicle’s decomposed Pauli intensity varies at different statuses, such as loading and heading. Therefore, a new metric, M, is needed to improve the vehicle detectability, and this is proposed as follows:

(8) $$M_{i}=\max\left(\left|x_{i}-E\left(x\right)\right|,\left|y_{i}-E\left(y\right)\right|,\left|z_{i}-E\left(z\right)\right|\right)$$

where $x_{i}$, $y_{i}$, and $z_{i}$ represent each Pauli decomposed component ${\left|\alpha \right|}^{2}$, ${\left|\beta \right|}^{2}$, and ${\left|\gamma \right|}^{2}$ at position i, respectively. The symbol E(⋅) denotes the ensemble average of road background on each Pauli component, *x*, *y*, and *z*. In the process of calculating the detectability using Formula (6), the signal intensity, S, corresponds to the maximum value among these three Pauli components at position i, and the background power is the mean value of the road’s M on the maximum Pauli component.

## 4. Experiments

Because the RCS of targets is affected by many factors, each experiment is undertaken to clarify only one factor by the means of keeping the other factors unchanged or similar. Those experiments are conducted in the order of size, aspect angle and shape for analysis of the detectability.

### 4.1. Experiment I

**Figure 2:**
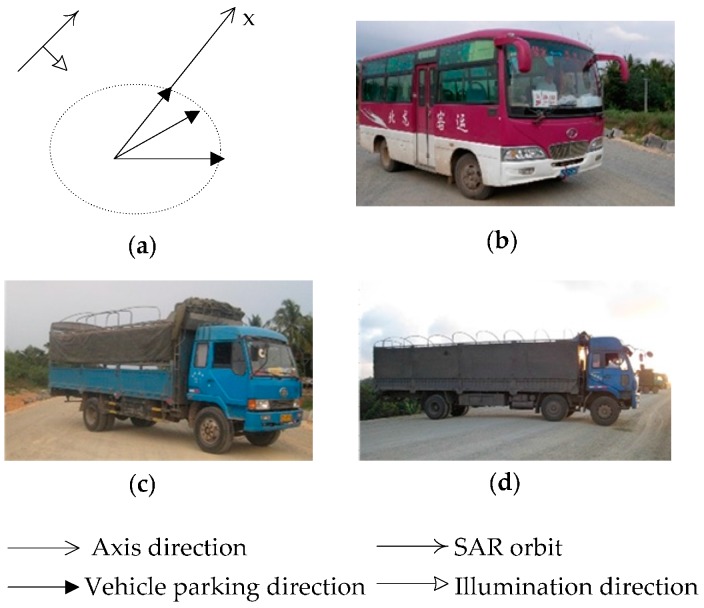
Vehicle deployment at three predetermined aspect angles in Experiment I: (**a**) the relationship between SAR and three predetermined aspect angles; (**b**) medium bus; (**c**) medium truck, and (**d**) larger truck.

To analyze the effect of a vehicle’s size on its detectability in the Radarsat-2 FP image, three types of vehicles, including medium buses, medium trucks, and larger trucks, were deployed at three typical aspect angles (0°, 45°, and 90°) in Experiment I. The deployment of each type of vehicle is presented in Figure 2.

### 4.2. Experiment II and III

**Figure 3:**
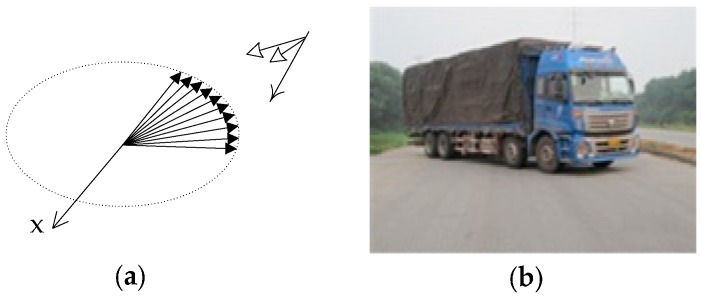
Ten trucks parked at continuous aspect angles and imaged by SAR in Experiment II and III at different incidence angles. (**a**) The geometric relationship between the SAR illumination direction and truck parking angle; and (**b**) a larger truck with a rectangular cuboid shape.

To evaluate the influence of varied aspect angles on detectability in Radarsat-2 FP images, the same type of larger truck was parked within the range of [180°, 275°] and imaged in Experiments II and III at different incidence angles. Figure 3 shows the distribution of the aspect angles.

### 4.3. Experiment IV

**Figure 4:**
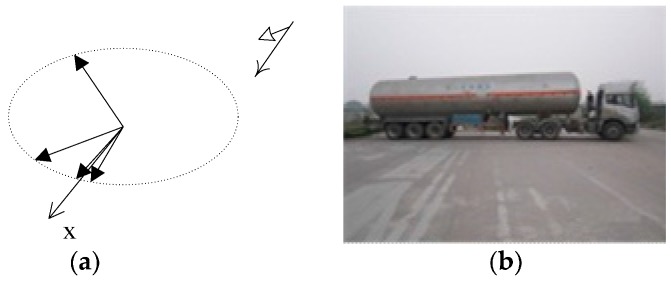
Four tank trucks parked at predetermined aspect angles in Experiment IV. (**a**) The geometric relationship between SAR and tank trucks; and (**b**) a tank truck with a cylindrical shape.

To assess the impact of a vehicle’s shape on detectability, four tank trucks at aspect angles of 2°, 45°, 151°, and 351° were imaged in Experiment IV. From the viewpoint of shape, a back-end cylindrical tank is significantly different from a parallelepiped truck, as shown in Figure 4.

## 5. Results and Discussion

**Figure 5:**
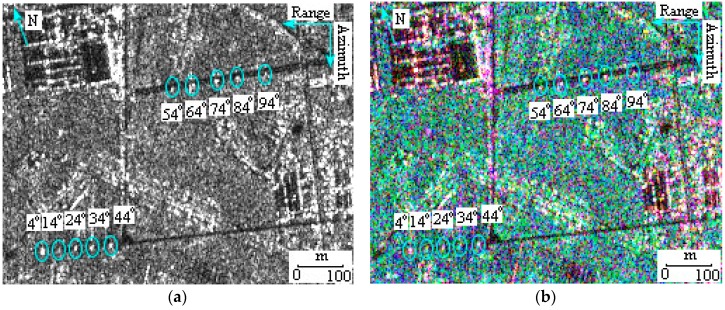
Ten larger trucks circled by cyan ellipses are parking at the consecutive intersection angles from 4° to 94° in FP image. (**a**) The total scattered power (SPAN) image; and (**b**) Pauli red-blue-green (RGB) composition image (Red: ${\left|\beta \right|}^{2}$, Green: ${\left|\gamma \right|}^{2}$, and Blue: ${\left|\alpha \right|}^{2}$).

Under the support of a geometric model of man-made objects, it is convenient to understand SAR images for backscattering analysis [20,21]. Taking into account the symmetry of the geometric models of the vehicles, the backscatter intensity is the same from both the left and right sides. For this reason, the scope of the aspect angle in this paper can be reduced from 360° to 180°. In the following discussion, the aspect angle is replaced by the minimum intersection angle in the range of [0°, 180°], which is the angle between the *x*-axis and the long side of the vehicle. Figure 5, as an example, is the FP image acquired in experiment III, in which ten larger trucks circled by cyan ellipses are parking at consecutive intersection angles. To quantitatively analyze the detectability under various influences, the vehicle intensities in SPAN and Pauli decomposed images are calculated using the aforementioned methods and applied to the following discussion.

### 5.1. Analysis of the Influence of the Vehicle’s Size on Detectability

**Figure 6:**
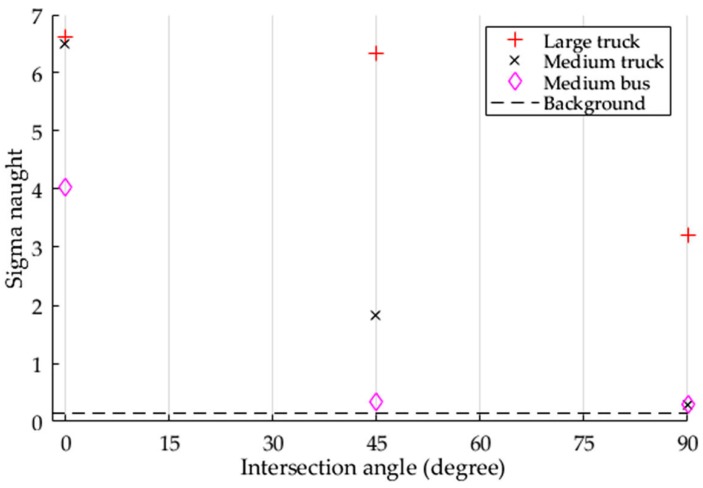
The sigma naught $\left(\sigma_{0}\right)$ of each vehicle at three intersection angles, as determined in Experiment I.

Three types of vehicles of different sizes were parked at the same intersection angles and imaged by SAR using the same imaging parameters in Experiment I. Figure 6 presents the maximum $\sigma_{0}$ of each vehicle at each intersection angle and the mean value of road background in the SPAN image.

Table 3 shows the value $P_{d}$ of each vehicle calculated using Formula (6), where the $P_{\mathrm{fa}}$ is first calculated using Formula (7) with the $x_{0}$ set as the intensity value of the medium truck at the intersection angle of 45°.

According to Table 3, a larger vehicle has a higher detectability than a smaller vehicle. This conclusion is based on the fact that the intensity results in this experiment are most likely only caused by the different vehicle sizes, since the other imaging parameters, such as shape, incidence angle, and intersection angle, are the same. In addition, all vehicles have a maximum intensity value at the intersection angle of 0°, where their long side is facing the illumination direction of the SAR. As the intersection angle increases, vehicle intensity in the SPAN image decreases due to the lessening of the areas projected on the perpendicular plane of the SAR illumination. The medium truck and bus have low detectability at the intersection of 90° because of their small head size and smooth top roof. Thus, it may also be concluded that it is difficult to obtain a higher value of $P_{d}$ at all intersection angles when the size of the parallelepiped-shaped vehicle is smaller than the SAR image resolution.

### 5.2. Analysis of the Influence of Intersection Angle on Detectability

**Figure 7:**
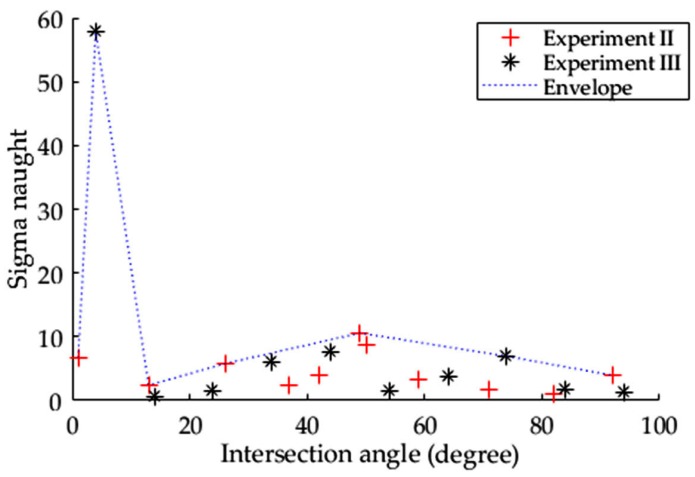
The $\sigma_{0}$ of larger trucks oscillates with increasing intersection angle, as determined in Experiments II and III.

In Experiments II and III, the $\sigma_{0}$ measurement of each vehicle at the corresponding intersection angle is marked in Figure 7. Investigating the changes of RCS over all of the intersection angles is an effective means to understand the mechanism of backscattering oscillation. This method is employed to interpret the backscattering fluctuation of buildings with varied orientation [22]. To clearly show the overall tendency of $\sigma_{0}$ with intersection angles, an envelope line covering all of the $\sigma_{0}$ values for each vehicle is drawn in Figure 7.

The main feature of Figure 7 is that the intensity of the vehicles oscillates with an increasing intersection angle. Following the envelope of $\sigma_{0}$, however, we can see that there are two peaks located near the intersection angles of 0° and 45°. This is due to the dihedral mechanism of SAR, which is formed between the vehicle side and the road near the intersection angle of 0°, and a natural trihedral mechanism of SAR is caused by the truck’s rear tail at approximately the intersection angle of 45°. Unfortunately, some trucks at approximately 45° do not have a high $\sigma_{0}$ value while they are loading because the trihedral corner at the rear tail facing the SAR is filled.

### 5.3. Analysis of the Influence of a Vehicle’s Shape on Detectability

**Figure 8:**
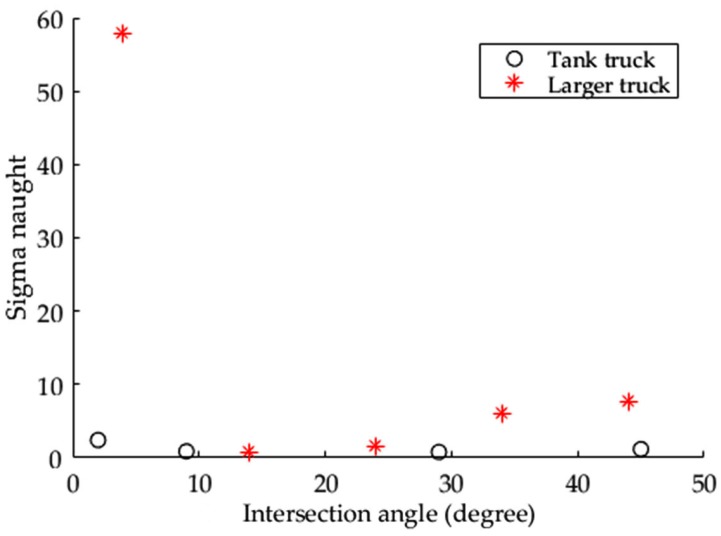
A comparison of $\sigma_{0}$ between cylindrical-shaped tank trucks in Experiment IV and parallelepiped-shaped larger trucks in Experiment III.

SAR images of tank trucks and larger trucks were acquired with similar incidence angles in Experiments III and IV, respectively. To compare their intensities at close aspect angles, the aspect angles of four tank trucks are reduced to 2°, 45°, 29°, and 9° in Figure 8 based on an assumption that the target with intersection angles D° and (180−D)° has equal backscattering intensity. The reason underlying this assumption is that the intensity at the intersection angle *D*° is caused by SAR illuminating from head to tail, which is similar to that resulting from illuminating in the opposite direction, i.e., an intersection angle (180−D)°.

The tank truck near intersection angle 0° has a higher intensity than those at the other three intersection angles. This phenomenon in Figure 8 is consistent with that in Figure 6 and Figure 7 for the other types of vehicles. Because the cylindrical-shaped tank trucks are bigger than the larger trucks, it is reasonable to expect that tank trucks may cause higher backscatter intensity. However, the intensity of the tank trucks is actually lower than that of the larger trucks at almost all intersection angles. This is mainly due to the crucial factor of shape, because the other parameters, such as incidence angle and aspect angle, are all similar. In detail, the smooth shape of the cylindrical tank has few areas to produce specular, double-bounce, and multiple reflections back to the SAR sensor. For the parallelepiped-shaped larger truck, however, the rough top roof creates diffuse backscattering during loading and forms a trihedral mechanism in the SAR when idling. In addition, the parallelepiped-shaped body on the road background forms a dihedral structure and constitutes more opportunities for backscatter to the SAR. Thus, it may be concluded that shape is a more crucial factor than size for producing higher detectability in SAR images.

### 5.4. Detectability Comparison between Different Metrics

**Figure 9:**
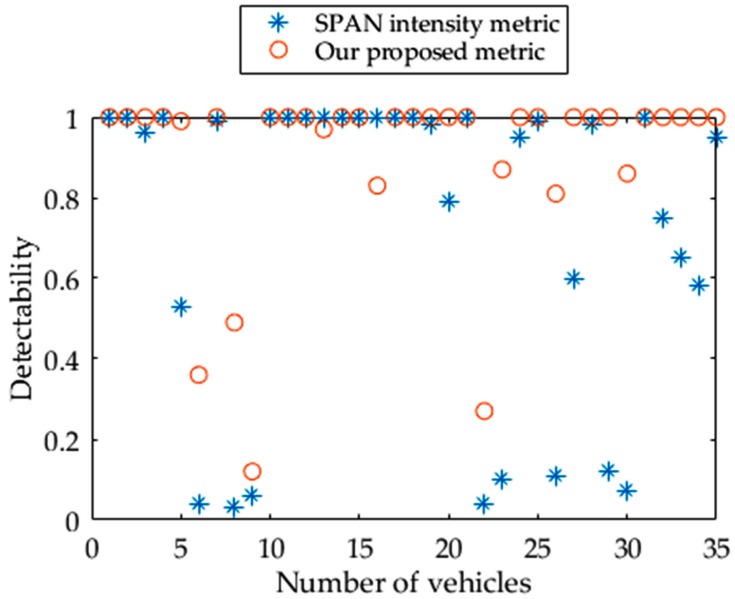
Comparison of detectability between SPAN intensity metric and our proposed metric.

CFAR detector is a well-known tool to detect objects in SAR images based on intensity metric, which has been widely applied to applications such as vessel and vehicle detection [12,18]. Figure 9 shows the detectability of all the vehicles in our experiments by using Formula (6), in which the same $P_{\mathrm{fa}}$ is employed. These values are calculated based on the SPAN intensity metric and our proposed metric (M), respectively.

Notably, the low detectability calculated by the SPAN intensity metric is significantly improved with the new metric, M, especially for vehicles with a small size. In detail, there are eight vehicles below the detectability of 0.5 using the SPAN intensity metric. In contrast, the number of vehicles with detectability less than 0.5 is reduced to four using the new M metric. Meanwhile, overall detectability is improved from 0.72 to 0.90 when the new metric is applied. In short, in comparison with the traditional SPAN intensity metric, the new metric is effective for improving vehicle detectability based on the polarimetric decomposition technique.

The reasons for the excellent performance achieved by this new metric can be analyzed as follows. For the process of Pauli decomposition, the boxcar filter is applied as a standing process to suppress the speckle intensity in single-look FP SAR images. Therefore, a small M value as the denominator in S/N is generated in the road background region, which is homogeneous and fits a normal distribution with stable statistic parameters [18]. In contrast, it is not feasible to predict which Pauli component is high around the vehicle region due to the vehicle’s heterogeneous characteristics. For the proposed metric to achieve a high value of S/N, the highest value among the three Pauli components at pixel position i is selected in Formula (8), and then the vehicle detectability is improved as the value of the numerator of S/N is increased.

**Figure 10:**
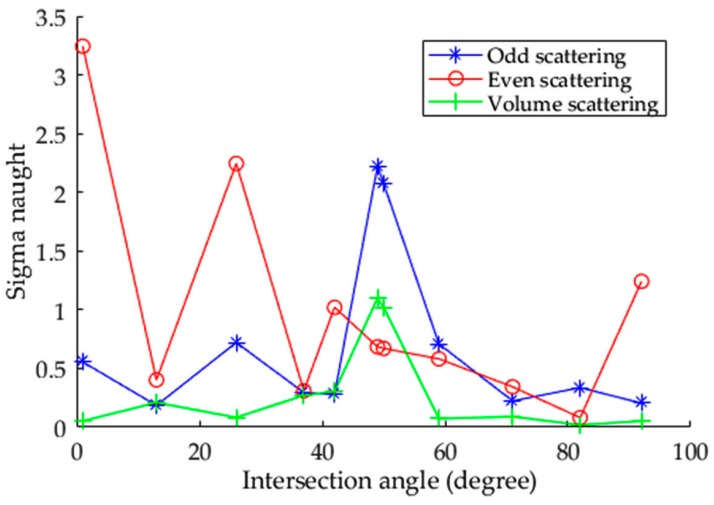
Each decomposed Pauli component of larger trucks oscillates with increasing intersection angle in experiment II.

Several similar metrics were also proposed for ship detection [23,24,25]. However, they are based on the same reasonable assumption that the ship is dominated by even-bounce scattering and line bounce scattering in fully polarimetric (FP) SAR images, while the sea is dominated by odd-bounce scattering [23,24]. Thus, those metrics select some certain decomposed polarimetric parameters to achieve excellent performance [25]. However, intensity oscillating with the change of aspect angle is the main characteristic of vehicle backscattering. Specifically, none of the decomposed Pauli components are dominant through the aspect angle range, as shown in Figure 10. Based on this fact, our new metric (M) is designed to automatically select the highest Pauli decomposed component value of a vehicle and, therefore, gets better performance of detectability.

## 6. Conclusions

To extend the application of SAR images for vehicle detection and monitoring of traffic congestion, in our experiments, images of four common types of vehicles in China with different sizes, shapes, and aspect angles were acquired with FP Radarsat-2. Then, vehicle characteristics were measured and analyzed. Their detectability was calculated and compared between two methods that were based on the SPAN intensity and decomposed polarimetric components.

Although the RCS is a function of combined parameters related to SAR imaging and vehicle, general conclusions based on the quantitative analysis of our experimental results can be drawn as follows. The significant characteristic of the RCS from the same type of vehicle is that it oscillates with the change of aspect angle. For different types of vehicles, however, the shape factor has more influence on the detectability than the size factor. In detail, from the viewpoint of the aspect angle factor, a vehicle has a high probability of detection when it is heading parallel to the direction of the SAR orbit. From the standpoint of the size factor, a small vehicle is difficult to detect if its size is smaller than that of the SAR image resolution. From the shape perspective, a vehicle that contains more canonical structures, such as dihedral and trihedral structures, has higher detectability than one with a smooth surface. In addition, detectability can be significantly improved using polarimetric decomposition for FP SAR images, whose performance is evaluated and validated by comparison between our proposed method and the traditional SPAN intensity method.

In addition, the vehicles used in our experiments are all electrically large objects in comparison to the wavelength of Radarsat-2. Since the Radarsat-2 wavelength at the C-band is longer than that of COSMO-SkyMed (COnstellation of small Satellites for the Mediterranean basin Observation) and TerraSAR-X at the X-band, it reasonable that our research results may be beneficial in the application of vehicle detection using COSMO-SkyMed and TerraSAR-X images.

## Figures and Tables

**Table 1:** Parameters of Radarsat-2 single-look fully polarimetric (FP) images in four experiments.

Experiment	Pixel Resolution (m)	Incidence Angle (°)	Orbit Direction	Beam Mode
I	4.7 × 4.9	23.2	Ascending	Q4
II	4.7 × 4.7	39.1	Descending	Q19
III	4.7 × 5.0	48.8	Descending	Q31
IV	4.7 × 5.0	48.2	Descending	Q30

**Table 2:** The size of each vehicle (m).

Vehicle	Length	Width	Height
Medium bus	5.7	2.2	2.6
Medium truck	8.7	2.3	3.4
Larger truck	11.9	2.5	3.8
Tank truck	15.8	2.5	4.0

**Table 3:** The detectability ($P_{d}$) of each vehicle at intersection angles of 0°, 45°, and 90° in Experiment I.

Vehicle	0°	45°	90°
Larger truck	0.999	0.999	0.962
Medium truck	0.999	0.534	0.039
Medium bus	0.993	0.032	0.060
